# Supplementary material for: Pregnant Mothers’ Medical Claims and Associated Risk of Their Children being Diagnosed with Autism Spectrum Disorder
Source: J Pers Med. 2021 Sep 24;11(10):950. doi: 10.3390/jpm11100950 (PMC8537202; doi:10.3390/jpm11100950)
Supplement: Supplementary file 1 [file jpm-11-00950-s001.zip › jpm-1277813-supplementary/Supplementary Files/Supplemental_Material_Figure_S1_Example_of_Variable_Thresholding.pdf]

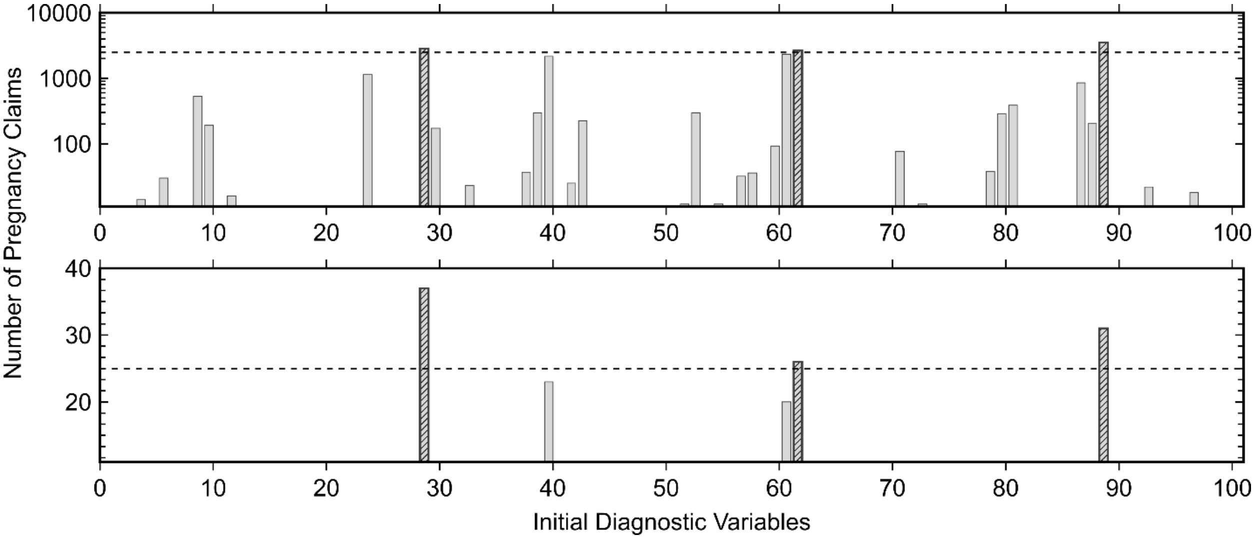

Figure S1. Example of variable thresholding. The number of claims is shown for the first 100 diagnostic variables within both cohorts (top) and only the ASD cohort (bottom). Dashed lines represent 2% threshold for both cohorts and ASD cohort, 2,477 and 25 claims, respectively. Variables with claims above the threshold (noted by patterned bars) are kept in the analysis. The y-axis begins at 11 in order to remain compliant with OptumLabs' de-identification policy.
