# Supplementary material for: Pregnant Mothers’ Medical Claims and Associated Risk of Their Children being Diagnosed with Autism Spectrum Disorder
Source: J Pers Med. 2021 Sep 24;11(10):950. doi: 10.3390/jpm11100950 (PMC8537202; doi:10.3390/jpm11100950)
Supplement: Supplementary file 1 [file jpm-11-00950-s001.zip › jpm-1277813-supplementary/Supplementary Files/Supplemental_Material_Table_S2_Highly_Correlated_Variables.pdf]

Table S2. Highly Correlated Variables

Variable	Category	P-value [1]	r [2] value
**Procedures Cardiovascular [3]**	**Procedural**	**1.8×10^{-2}**	0.95
Surgical Procedures Cardiovascular System	Procedural	2.2×10^{-2}	
Need for Prophylactic Vaccination against Viral Diseases	Diagnostic	6.5×10^{-5}	0.88
**Vaccinations [3]**	**Procedural**	**4.5×10^{-6}**	
Diagnostic Agents	Pharmacy	1.8×10^{-2}	0.88
**Durable Medical Equipment Diabetic[3]**	**Pharmacy**	**2.8×10^{-3}**	
Immunization Administration for Vaccines Toxoids	Procedural	1.3×10^{-5}	0.86
**Vaccinations [3]**	**Procedural**	**4.5×10^{-6}**	
Need for Prophylactic Vaccination against Viral Diseases	Procedural	6.5×10^{-5}	0.80
Immunization Administration for Vaccines Toxoids	Diagnostic	1.3×10^{-5}	
**Acquired Hypothyroidism [3]**	**Pharmacy**	**5.3×10^{-2}**	0.80
Endocrine Thyroid Therapy	Diagnostic	1.8×10^{-1}	

[1] p-value was calculated using unadjusted logistic regression analysis.
[2] Pearson correlation coefficient.
[3] bolded variables are kept in the logistic regression analysis.
